# Beyond the Classic Latissimus Dorsi Flap: A Decision-Making Algorithm for Technique Selection in Complex Breast Reconstruction

**DOI:** 10.3390/jcm15093500

**Published:** 2026-05-02

**Authors:** Federico Lo Torto, Lorenzo Santarelli, Donato Casella, Federico Tamborini, Ferruccio Paganini, Paolo Noccioli, Marco Marcasciano

**Affiliations:** 1 Plastic Surgery Unit, Santo Spirito Hospital, Department of Medical, Oral and Biotechnological Sciences, “G. d’Annunzio” University of Chieti-Pescara, 66100 Chieti, Italy; 2 Plastic Surgery Unit, Department of Surgery, Sapienza University of Rome, 00161 Rome, Italy; 3 Plastic and Reconstructive Surgery Unit, Molinette Hospital, Department of Surgical Sciences, University of Turin, 10126 Turin, Italy; 4 Division of Plastic and Reconstructive Surgery, Department of Biotechnology and Life Sciences, University of Insubria, 21100 Varese, Italy; 5 Breast Surgery Unit, Santo Spirito Hospital, 65124 Pescara, Italy; 6 Plastic and Reconstructive Surgery Unit, Department of Experimental and Clinical Medicine, Magna Graecia University, 88100 Catanzaro, Italy

**Keywords:** latissimus dorsi flap, breast reconstruction, hybrid breast reconstruction, extended latissimus dorsi flap, fat-augmented latissimus dorsi flap, KISS flap, salvage breast reconstruction, reconstructive algorithm, irradiated breast

## Abstract

**Background**: The latissimus dorsi (LD) flap remains a reliable option for breast reconstruction in irradiated, salvage, and non-microsurgical candidates. Despite the availability of multiple LD-based variants, practical guidance for technique selection remains limited. **Methods**: We retrospectively analyzed 40 consecutive LD-based breast reconstructions performed over 5 years at two Italian centers. Cases were categorized as classic LD, hybrid LD, extended LD, V-FALD, or KISS flap. Clinical variables, reconstructive setting, complications, and reoperations were described, and a decision-making algorithm was derived from institutional practice and literature integration. **Results**: The cohort was characterized by a high-complexity profile, with 36/40 patients (90%) exposed to radiotherapy and 22/40 (55%) treated in a salvage setting. Hybrid LD was the most frequently used technique, accounting for 23/40 cases (57.5%), followed by KISS flap in 6/40 (15%), extended LD in 5/40 (12.5%), V-FALD in 4/40 (10%), and classic LD alone in 2/40 (5%). Technique selection was primarily driven by skin-envelope adequacy, breast volume requirement, and the feasibility of a fully autologous reconstruction. Major complications occurred in 2/40 patients (5%), revision surgery occurred in 3/40 (7.5%), and no total flap necrosis was observed. Donor-site morbidity occurred in 10/40 (25%) of cases and was managed conservatively. **Conclusions**: LD-based breast reconstruction should be viewed as a versatile reconstructive platform rather than a single technique. A pragmatic algorithm may support surgical planning and help tailor the most appropriate LD variant to defect characteristics and reconstructive goals.

## 1. Introduction

The latissimus dorsi (LD) flap remains one of the most established options in breast reconstruction. First described in the early twentieth century and incorporated into modern breast reconstruction during the 1970s, it has maintained a central role because of its reliable vascular anatomy, technical reproducibility, and broad applicability across immediate, delayed, and salvage settings [1,2]. Although microsurgical abdominal flaps are often regarded as the reference standard for autologous reconstruction, the pedicled LD flap continues to represent a dependable reconstructive workhorse [2,3], particularly in patients who are poor candidates for lengthy microsurgical procedures or who require a more adaptable solution to complex defects.

Its contemporary relevance is especially evident in challenging clinical scenarios. The LD flap can bring healthy, well-vascularized tissue into an irradiated or scarred recipient bed [4,5,6], improve soft-tissue coverage in implant-based reconstruction, and serve as a salvage option after failure of previous prosthetic reconstruction. In irradiated breasts, the addition of vascularized autologous tissue may improve contour, soften contracted tissues, and reduce exposure-related problems, which explains why LD-based reconstruction has retained a specific indication in this setting even in the era of perforator flaps.

A further reason for the renewed interest in LD-based reconstruction is the progressive expansion of its technical variants. Beyond the classic flap, surgeons now have access to hybrid reconstructions with implant or expander support (Hybrid LD), extended harvests designed to maximize autologous volume (Extended LD), and fat-augmented strategies such as FALD/V-FALD aimed at avoiding implants altogether in selected patients [7,8,9,10,11,12,13]. More recently, the KISS LD flap has been described to increase available skin and volume [14,15], thereby extending the role of the pedicled LD flap in fully autologous reconstruction and in defects characterized by significant skin-envelope deficiency.

Despite this versatility, the literature still offers limited practical guidance on how to select the most appropriate LD variant for a given patient [3,12,16,17]. Most studies focus on individual techniques, specific indications, or single-institution experiences, while a simple decision-making framework integrating skin-envelope status, required breast volume, radiotherapy, and the feasibility of a fully autologous reconstruction remains insufficiently defined. The aim of the present study was therefore to review our 5-year dual-center experience and to propose a pragmatic surgical algorithm to support technique selection in everyday clinical practice.

## 2. Materials and Methods

This study was designed as a retrospective, dual-center observational case series including 40 consecutive LD-based breast reconstructions performed over a 5-year period at two Italian centers: Policlinico Umberto I, Rome, and Ospedale Santo Spirito, Pescara. The purpose of the study was to analyze our institutional use of the latissimus dorsi flap across different reconstructive scenarios and, on the basis of both clinical experience and available evidence, to derive a pragmatic decision algorithm for technique selection.

All patients undergoing breast reconstruction with an LD-based strategy during the study period were included. Reconstructions were categorized according to the operative technique used: classic LD flap alone, hybrid reconstruction with implant or tissue expander support, extended LD flap, Vertical Fat-Augmented Latissimus Dorsi flap (V-FALD), and KISS LD flap. In our practice, the indication for an LD-based reconstruction was considered mainly in the presence of previous or planned radiotherapy, salvage after failure of a previous reconstructive procedure, patient preference for an autologous approach, absence of an adequate abdominal donor site, previous abdominal surgery, obesity, smoking, or when microsurgical reconstruction was considered unsuitable or unavailable.

All reconstructions included in this series were performed through standard open harvest techniques. No endoscopic or robot-assisted latissimus dorsi flap harvests were performed during the study period. In hybrid reconstructions, no acellular dermal matrix was used; implant or tissue expander coverage relied on the latissimus dorsi flap itself, according to institutional practice.

Preoperative planning was based on the reconstructive setting, the condition of the recipient site, the expected or actual quality of the residual skin envelope, the anticipated or already present volume deficiency, and the feasibility of a totally autologous reconstruction. In immediate cases, skin deficiency and volume requirements were estimated according to preoperative breast features and anticipated oncologic resection, whereas in delayed and salvage reconstructions, they were assessed based on the actual local condition. Dorsal donor-site availability was clinically evaluated, including pinch testing, to estimate the amount of recruitable soft tissue. For practical purposes, breast volume requirement was categorized according to the estimated weight of the breast volume to be addressed during reconstruction: small (<300 g), medium (300–500 g), and large (>500 g). This classification was used as a pragmatic planning tool and reflected the overall reconstructive requirement in each case, considering preoperative breast characteristics, contralateral breast features, skin-envelope condition, and the expected or actual extent of the defect. Major skin-envelope deficiency directed the reconstructive plan toward techniques capable of restoring both skin and volume, particularly the KISS flap, whereas cases with an adequate skin envelope were further stratified according to breast size and anticipated volume deficiency. When required to achieve overall breast balance, contralateral symmetrization was considered part of the reconstructive strategy rather than a secondary corrective procedure and could be performed either during the index operation or in a staged fashion.

For each patient, a standardized core dataset was collected, including age, body mass index, smoking status, prior or planned radiotherapy, reconstructive setting (immediate, delayed, or salvage), degree of skin deficiency, estimated breast volume requirement, operative technique, major complications, revision surgery, and follow-up. All variables were extracted from prospectively maintained operative and clinical records; no missing data were identified for the predefined core variables. Major complications were defined as events requiring unplanned surgery, implant removal, or major deviation from the initial reconstructive pathway. Revision surgery was defined as any additional operative procedure performed to improve or complete the reconstruction.

The primary objective of the study was not to compare the different techniques statistically, but to identify recurrent clinical decision patterns and formalize them into a practical algorithm. For this reason, the analysis was primarily descriptive. Continuous variables were summarized using median and range, while categorical variables were reported as frequencies and percentages. The proposed algorithm was then structured by integrating the decision criteria observed in our series with the indications, technical limits, and outcomes reported in the current literature on classic, hybrid, extended, fat-augmented, and KISS LD reconstructions. Clinical photographs, when presented, were used as illustrative examples of the main reconstructive scenarios addressed by the algorithm.

## 3. Results

A total of 40 LD-based breast reconstructions were included. The cohort reflected a predominantly high-complexity population: median age was 52 years (range, 42–64), median body mass index was 26.9 kg/m^2^ (range, 21.9–33.1), 13/40 patients (32.5%) were active smokers, and 36/40 patients (90%) had received or were scheduled to receive radiotherapy. Most reconstructions were performed in a salvage setting (22/40, 55%), whereas 14/40 cases (35%) were immediate and 4/40 (10%) were delayed.

**Figure 1:**
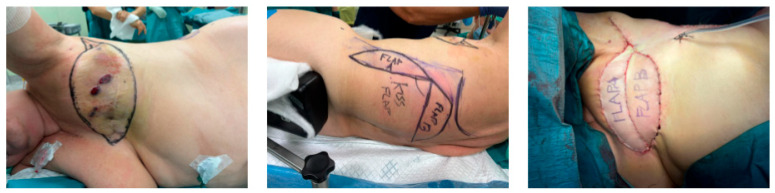
Intraoperative planning and reconstruction with the KISS latissimus dorsi flap. The sequence shows the recipient-site defect, preoperative marking of the double skin paddle, and final inset after flap transfer.

**Figure 2:**
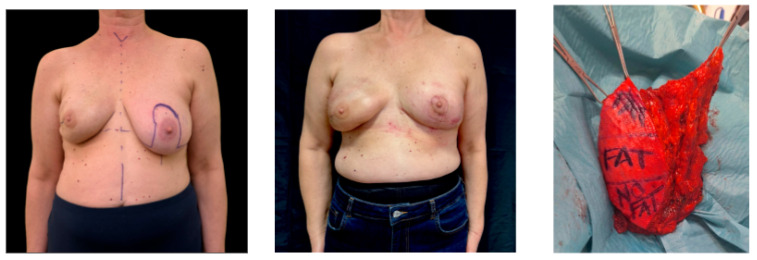
Representative case of breast reconstruction with the V-FALD flap. The sequence shows preoperative planning, postoperative frontal view, and intraoperative appearance of the harvested flap.

**Figure 3:**
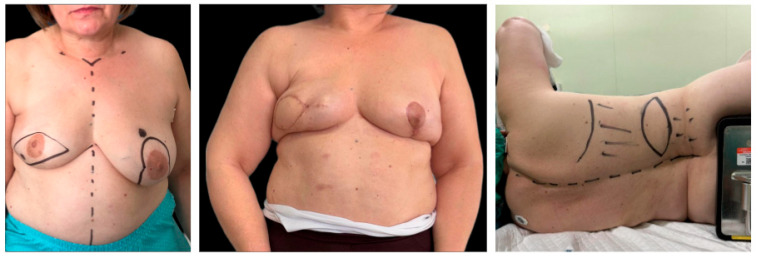
Representative case of classic latissimus dorsi flap breast reconstruction. The sequence shows preoperative marking of the recipient site, postoperative result, and design of the dorsal donor site. The patient is scheduled to undergo secondary lipofilling to further optimize contour and volume.

Regarding technique distribution, hybrid LD reconstruction was the most frequently adopted strategy, accounting for 23/40 cases (57.5%). This was followed by the KISS LD flap in 6/40 (15%), extended LD flap in 5/40 (12.5%), V-FALD flap in 4/40 (10%), and classic LD flap alone in 2/40 (5%). Breast volume was classified as small in 4/40 cases (10%), medium in 22/40 (55%), and large in 14/40 (35%). Severe skin-envelope deficiency was documented in 6/40 patients (15%). Representative clinical examples of these reconstructive scenarios are provided in Figure 1, Figure 2 and Figure 3.

**Figure 4:**
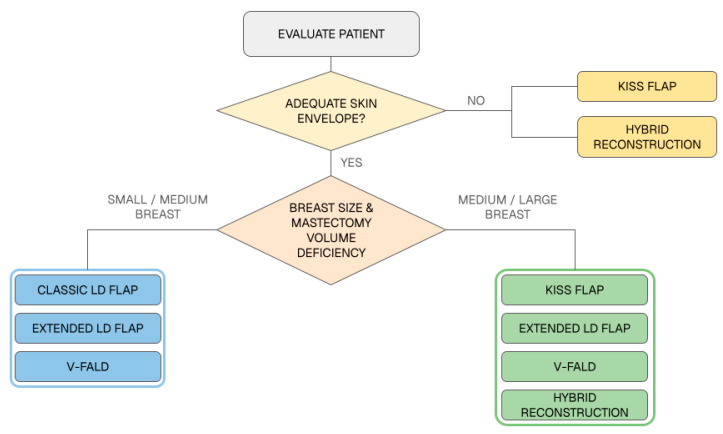
Proposed algorithm for technique selection in latissimus dorsi-based breast reconstruction. Surgical choice is guided by skin-envelope adequacy, breast volume requirement, and the feasibility of a fully autologous reconstruction. LD, latissimus dorsi; V-FALD, vertical fat-augmented latissimus dorsi.

Three main determinants guided technique selection: adequacy of the skin envelope, required breast volume, and the feasibility of a fully autologous reconstruction. In the context of the proposed decision-making algorithm (Figure 4), an adequate skin envelope was defined as a residual skin envelope sufficient to avoid major cutaneous replacement, even when a limited or moderate skin deficiency was present. As summarized in the algorithm, classic LD alone was reserved for small-volume reconstructions in immediate or salvage settings. Hybrid LD reconstruction predominated in patients requiring medium- to large-volume replacement when dorsal tissue alone was considered insufficient to achieve a fully autologous reconstruction with adequate shape and projection. Extended LD and V-FALD, however, were not restricted to small- or medium-volume breasts and were also used in selected medium- to large-volume reconstructions in patients with favorable body habitus, sufficient dorsal tissue, and a preference to avoid implants. In our practice, the extended LD flap was preferred when greater cutaneous coverage was needed, whereas V-FALD was favored when volumetric enhancement was the main reconstructive requirement. The primary indication for the KISS flap was the reconstruction of large skin defects, given its ability to provide substantial skin replacement together with autologous tissue transfer. However, in selected cases classified within the algorithm as having an adequate skin envelope, the KISS flap could also be used for predominantly volumetric defects after deepithelialization, when additional autologous tissue and shaping were required.

Postoperative outcomes were acceptable despite the complexity of the cohort. Major complications occurred in 2/40 patients (5%): one patient developed a recipient-site infection requiring unplanned surgical treatment, whereas another experienced partial skin paddle compromise requiring operative management. Revision surgery was required in 3/40 cases (7.5%), including two lipofilling procedures and one secondary correction for asymmetry. No total flap necrosis was observed. Donor-site morbidity occurred in 10/40 patients (25%) and consisted exclusively of seroma or delayed wound healing, the latter being documented in 3/40 patients (7.5%); all donor-site complications were managed conservatively, with no need for surgical intervention. Median follow-up was 17 months (range, 10–29).

## 4. Discussion

The present study supports the concept that the latissimus dorsi flap should be regarded less as a single reconstructive procedure and more as a versatile reconstructive platform. In our series, most patients belonged to categories traditionally considered challenging for breast reconstruction, namely irradiated cases, salvage reconstructions, smokers, and patients in whom microsurgical abdominal flaps were unsuitable or unavailable. This profile is fully consistent with contemporary literature, which continues to identify the pedicled LD flap as a reliable option in complex settings because of its robust vascularity, technical reproducibility, and ability to transfer healthy tissue into compromised recipient beds [4,5,12].

Hybrid LD reconstruction represented the predominant reconstructive strategy in our cohort, a finding that reflects the persistent clinical relevance of implant-assisted pedicled reconstruction in complex breast defects. In routine practice, the classic flap often provides excellent soft-tissue coverage and improves the quality of the reconstructive bed, but may not consistently provide sufficient volume and projection in patients with medium- or large-sized breasts [18]. In this setting, the addition of an implant or tissue expander remains particularly useful when native tissue is insufficient and an adequate breast volume must still be achieved. At the same time, the literature suggests that implant-assisted LD reconstruction may be associated with lower patient-reported satisfaction than fully autologous LD reconstruction and remains exposed to implant-related complications, particularly in irradiated patients [12,19]. For this reason, the hybrid approach remains valuable in our algorithm, but mainly when the available dorsal tissue is insufficient to achieve an autologous reconstruction with acceptable volume and shape.

A relevant aspect of our algorithm is that extended LD and V-FALD are not restricted to small- or medium-sized breasts. In selected patients with favorable body habitus, adequate dorsal soft tissue, and a strong preference to avoid implants, both options can be extended to medium-large volume reconstructions [20,21]. This point is particularly evident for the extended LD approach. In a previous study, some of the authors of the present manuscript reported that the ELD-K flap could expand the role of total autologous pedicled reconstruction in medium- and large-breasted patients by recruiting additional tissue and improving projection [8]. The rationale of the present algorithm is consistent with that prior experience. Similarly, fat-augmented LD strategies have progressively expanded the autologous potential of the flap, and recent FALD series [9,10,22] confirm that careful volume planning, intraoperative shaping, and immediate fat transfer can make single-stage autologous reconstruction feasible beyond very small breasts in selected cases.

The role of the KISS flap extends beyond merely addressing severe skin deficiency [14,15,23,24]. While major skin-envelope shortage remains a primary indication due to the flap’s capacity to transfer substantial skin and soft tissue, its utility is broader. In patients with a preserved skin envelope requiring primarily volumetric restoration and shaping—including selected medium-to-large breasts—the flap can be deepithelialized. This expanded application aligns with both the original KISS flap concept and the experience of Brunetti et al. [15], who reconstructed small, medium, and large breasts without implants, reserving fat grafting strictly for secondary refinement rather than primary volume creation. Consequently, the KISS flap holds an intermediate position in our algorithm: it is the treatment of choice for significant skin replacement, but it also serves as an effective, fully autologous, volume-enhancing strategy when the skin envelope is adequate.

Another point emerging from our series is the acceptable safety profile despite the complexity of the cohort. The rates of major complications and revision surgery were low, even though most patients were irradiated, and a substantial proportion underwent salvage reconstruction. These findings should be interpreted cautiously, but they align with the broader literature suggesting that LD-based breast reconstruction remains reliable and reproducible across immediate, delayed, and salvage settings [2,3,11]. Systematic and narrative reviews have also shown generally favorable patient-reported outcomes, with autologous LD techniques often performing particularly well in satisfaction domains when compared with implant-assisted variants [16,20]. Taken together, these data support the practical role of the LD flap not necessarily as a replacement for all microsurgical options, but as a dependable strategy in real-world situations where defect characteristics, patient factors, or institutional resources limit other reconstructive pathways.

From a practical standpoint, the main contribution of this study lies in the attempt to translate this versatility into a simple decision framework. Our data suggest that three variables drive most of the reconstructive choice: the adequacy of the residual skin envelope, the required breast volume, and the realistic feasibility of a completely autologous reconstruction. Once these are established, the algorithm becomes more intuitive: classic LD for very small breasts, hybrid LD when prosthetic support is required, extended LD or V-FALD when preserved skin and sufficient dorsal volume make autologous reconstruction feasible, and KISS flap when major skin replacement is necessary or when a larger autologous flap is needed for selected medium-large breasts. In this sense, the algorithm is not intended to rigidly categorize techniques, but rather to organize them within a reproducible clinical logic.

This study has several limitations that should be acknowledged. First, it reflects the experience of two centers and a relatively limited number of patients, which inevitably reduces the generalizability of the findings. Second, the different LD variants were applied to different reconstructive scenarios rather than to comparable groups, so the study should not be interpreted as a head-to-head comparison among techniques. Third, although the retrospective design is appropriate for describing practice patterns and generating a pragmatic algorithm, it does not allow the same level of control as a prospective comparative study. In addition, patient-reported outcomes were not collected in a standardized fashion across the entire cohort, which limits the possibility of correlating the algorithmic choice with subjective satisfaction. Further prospective, multicenter studies will be necessary to validate the proposed algorithm and confirm its generalizability across different patient populations and surgical settings. Nonetheless, we believe these limitations do not undermine the clinical value of the study. On the contrary, the strength of this series lies in representing a real-world population of complex reconstructions and in formalizing a practical approach that can be tested, refined, and externally validated in future multicenter studies.

## Data Availability

The data presented in this study are available on request from the corresponding author. The data are not publicly available due to privacy and ethical restrictions.
